# The relationship of syntactic complexity and rhetorical move-steps in research article discussions: A comparative analysis of Chinese and native English writers

**DOI:** 10.1371/journal.pone.0334860

**Published:** 2025-10-23

**Authors:** Yuan Zhang, Zewen Wang

**Affiliations:** School of Foreign Languages, Shandong Normal University, Ji’nan, Shandong Province, China; Bahir Dar University, ETHIOPIA

## Abstract

This study seeks to identify the differences in syntactic complexity (SC) across rhetorical move-steps in the discussion section of research articles (RAs) written by Chinese and native English writers. The corpus consisted of 200 RA discussion sections from the field of Applied Linguistics. Each sample was manually annotated for rhetorical move-steps using the seven-move framework of RA discussion proposed by Yang and Allison (2003). The results revealed that distinct SC patterns across rhetorical move-steps, with differences most pronounced at the phrasal-level complexity. Chinese writers demonstrated greater SC performance in M2 *Reporting results*, M4S1 *Interpreting results*, M4S2 *Comparing results with literature*, and M4S3 *Accounting for results*, characterized by compressed noun phrases (NPs) through significantly more frequent use of pre-modifier sequences and multiple-level prepositional phrases. In contrast, native English writers presented greater SC in M3 *Summarizing results*, M4S4 *Evaluating results*, M5 *Summarizing the study*, M6S2 *Indicating significance/advantage*, M7S1 *Making suggestions*, and M7S2 *Recommending further research*, distinguished by more extensive use of complex NPs with both phrasal and clausal modifiers. Implications for L2 academic writing practice and pedagogy are discussed.

## Introduction

Over the past few decades, syntactic complexity (SC) has long been regarded as an important construct in second language (L2) writing research, offering valuable insights into L2 development and writing proficiency [1–4]. As one of the key dimensions of linguistic complexity, SC refers to the variety, sophistication, and elaboration of syntactic structures manifested in language production, and has been shown to be a reliable indicator of L2 writing quality, proficiency, and development [5–11]. In the context of English for academic purposes (EAP) writing, SC serves as a vital lens for examining how writers mobilize syntactic resources to construct meaning and fulfill the communicative demands of academic discourse.

A substantial body of research has identified multiple factors influencing SC performance in L2 academic writing, including the first language (L1) backgrounds [7,12–14], language proficiency [3,6], and rhetorical demands of different genres [15,16]. In recent studies, scholars have shifted focus toward micro-level genre structures, i.e., rhetorical move-steps, and examined how SC performance varies across these structures within particular writing genres [17], highlighting the potential of integrating rhetorical structures with formal syntactic features to bridge the “function-form gap” in EAP writing [18,19]. While notable progress has been made in exploring cross-disciplinary variation in SC associated with different rhetorical move-steps [20,21], relatively little attention has been paid to whether SC serving similar rhetorical purposes also varies across writers with different L1 backgrounds.

The L1 background of writers matters because it shapes writers’ preferred syntactic structures and these preferences can influence how rhetorical functions are realised within a given genre. Contrastive rhetoric research has shown that L1 rhetorical patterns and discourse norms significantly affect how writers organize their arguments and interpret genre conventions [22,23]. For example, writers from languages with hypotactic traditions may prefer using embedded clauses to convey argumentation, while those from more paratactic traditions may lean more on coordination and shorter clauses.

In the research article (RA) writing, the discussion is “an integral part in the writing process” of the article, serving as the stage where authors interpret, evaluate, and substantiate their findings. In Swales’ (1990) communicative-moves framework, the discussion functions as the pivotal space where writers step back and take a broad look at the findings as a whole. A compelling discussion section is often seen as a key determinant of an RA’s success. However, it presents a considerable challenge, especially for those internationally oriented L2 writers who not only need to overcome linguistic barriers but also adapt to academic writing norms and conventions that differ from those in their native academic communities. However, there remains a scarcity of studies that focus specifically on the RA discussion writing by L2 writers.

Therefore, to address this research gap, the present study investigates SC across rhetorical move-steps in RA discussion section, with a specific focus on cross-linguistic variation between Chinese and native English writers. By examining how writers from different linguistic backgrounds deploy syntactic resources to achieve rhetorical objectives, the study aims to offer insights that are both theoretically significant and pedagogically valuable for enhancing L2 academic writing instruction.

## Literature review

### Syntactic complexity research of RA part-genres

SC, defined as the degree of variation, sophistication, and elaboration of the syntactic structures used in language production [3,24–26], has received considerable attention in L2 writing research. Conceptualized as a multi-dimensional construct encompassing global, clausal and phrasal-level complexity [27], SC can be examined by utilizing either large-grained indices (length or ratio-based measures) [2,24,25] or fine-grained indices (frequency-based measures of individual syntactic structures) [7,13,14,21,28–30].

Many previous studies have investigated factors influencing SC performance in L2 academic writing, and demonstrated that genre is one of the crucial determinants. For example, Lan and Sun [31] compared the use of 11 noun phrase (NP) modifiers in argumentative essays written by Chinese writers and published RAs. They found that the argumentative essays exhibited lower usage frequencies across all 11 NP modifiers compared to the RAs. In addition, other studies have focused on variations of SC across different RA part-genres. For instance, Kang, Lu, and Zhang [32] and Liu and Li [33] compared SC between RA abstracts and lay summaries, which are two different kinds of research summaries that distill the key contents of RAs for readers. Despite focusing on different disciplines (medicine and marine science, respectively), their findings converged to reveal similar genre-related patterns: lay summaries exhibited shorter production units (MLS, MLT and MLC), but a greater usage of clausal subordination (C/S, DC/C) compared to RA abstracts. Casal, Lu, Qiu, Wang, and Zhang [15] investigated SC performance across four RA part-genres (i.e., introduction, method, results, and discussion), and found that the discussion section was significantly more syntactically complex than the results and methods section. Following this research strand, Yin, Gao, and Lu [16] further compared seven different part-genres (i.e., abstract, introduction, literature review, method, results, discussion, and conclusion) of applied linguistic RA, and found that SC patterns significantly vary across different RA sections. Their study reported that RA discussion section exhibited significantly more usage of subordinate clauses and verb phrases than method section, and more usage of verb phrases but less complex nominals and coordinate phrases than abstract section. These studies, to a large extent, have deepen our understanding of the syntactic features and variations across RA part-genres [34]. However, existing SC research on RA part-genres has primarily focused on the SC patterns of cross-part-genre variation from a disciplinary perspective, and little attention has been paid to investigate cross-linguistic SC variations within the same part-genre. This remains an important research gap and warrants further empirical investigation.

### Syntactic complexity research of Chinese EFL writers

Numerous studies have found salient syntactic patterns of English RAs written by Chinese English as foreign language (EFL) writers. It was reported that Chinese EFL writers tend to exhibit a heavy reliance on coordination, while making comparatively less use of subordination in their L2 academic writing. For example, Song and Wang [35] compared the English abstracts of doctoral dissertations written by Chinese and native English PhD students. Their results revealed that Chinese students used complex nominals, coordinated phrases and clauses significantly more frequently, but employed dependent clauses significantly less often than their native English counterparts. These differences were attributed to their distinct language acquisition environments. Their findings partially align with those of Xue and Ge [36], who found that the SC of RA abstracts written by Chinese writers was primarily contributed by the frequent use of coordinated phrases. However, the tendency for Chinese writers to favor coordinated structures does not appear to be consistent across all genres. For instance, in a comparative study of argumentative writing written by Chinese and native English college students, Lu and Ai [37] found that Chinese students used significantly fewer coordinated sentences than their native English peers.

Furthermore, with regard to phrasal complexity, Ruan [14] identified distinct patterns of phrasal complexity in RA abstracts authored by Chinese and native English writers. Specifically, Chinese writers were found to rely more heavily on using pre-modifiers in constructing NPs, such as nouns and multiple noun sequences as modifiers, whereas native English writers made greater use of post-modifiers, particularly prepositional phrases. This divergence may be attributed to language transfer [36]. Meanwhile, Chinese writers also exhibited a preference for using phrasal modifiers in their sub-modifications, reflecting a strong reliance on lexical knowledge. In contrast, native English writers were more likely to “draw on their repertoire of syntactic knowledge to convert clausal structures into phrasal premodifications” (p. 45), thereby displaying far more diverse and productive modifying patterns compared to Chinese writers.

These distinctive syntactic features observed in Chinese writers’ L2 academic writing can also be interpreted through the lens of the “constrained language” framework. According to Kruger and Van Rooy’s definition [38], L2 academic writing constitutes a type of constrained language which is produced in communicative contexts characterized by conspicuous cognitive and social constraints. Many studies showed that such constraints, particularly those arising from the influence of a writer’s first language, significantly shape language usage [39–41], and provide evidence that constrained writing varieties have a lower SC than those non-constrained [39,42]. Many studies examined the distinct syntactic characteristics influenced by Chinese language background in their English as second language (ESL) writing [39,42]. For instance, Chen, Li and Liu [39] examined the SC of Translated English (TE) and EFL news articles by Chinese writers using 14 holistic syntactic measures, with native English (NE) texts serving as the benchmark. They found that both TE and EFL texts exhibit higher mean length of clause (MLC) and T-unit (MLT), as well as a marked preference for using coordinate structures (CP/C and CP/T) compared to NE texts. This heightened use of coordination is attributed to a typological preference for parataxis in Chinese [43]. Conversely, these texts demonstrated lower overall sentence complexity and a reduced degree of subordination, pointing to a general simplification phenomenon in both translated and EFL writing. These results corroborate the findings of Liu and Afzaal [42], who reported that translated texts made by Chinese translators exhibit reduced SC compared to non-translated texts, especially in terms of subordination and overall sentence complexity. Moreover, from a phrasal perspective, Liu, Fang and Wei [44] identified the frequent use of left-branching structures in NPs as a distinctive feature of Chinese L2 writing. This pattern aligns with Ruan’s [14] findings and is likely attributable to language transfer from Chinese.

Existing studies revealed a range of distinctive SC features in L2 writing produced by Chinese writers, suggesting that Chinese L2 writers tend to adopt different linguistic strategies in realizing genre conventions and rhetorical strategies in L2 academic writing, likely shaped by deep-rooted L1 cognitive and typological influences. Given these distinctive features, further investigating Chinese writers’ L2 academic writing is particularly meaningful, as it can offer valuable insights into how L1 background interacts with rhetorical demands in shaping syntactic choices in academic discourse.

### Syntactic complexity in rhetorical move-steps

Recent studies have increasingly concentrated on the syntactic realization of rhetorical functions in L2 academic writings [29], where scholars have investigated how various syntactic structures respond to rhetorical demands in academic writing and the SC used across internal rhetorical structures [16,45]. As noted by Casal et al. [15], each RA section exhibits distinct patterns of SC, influenced by its rhetorical objectives and communicative purposes. For example, Lu, Casal, and Liu [30] analyzed SC across rhetorical move-steps in the introduction section of social science RAs, and found that SC measures, particularly global and phrasal indices, significantly different across rhetorical move-steps.

Following this research strand, Lu et al. [29] further examined SC variations across rhetorical structures from a disciplinary perspective. By utilizing a corpus of RA introductions from two social science disciplines (i.e., anthropology and sociology) and engineering disciplines (i.e., chemical and electrical engineering), the study reported significant SC variation both across disciplines and across rhetorical move-steps, highlighting that the SC of RA introductions is shaped jointly by disciplinary conventions and rhetorical purposes.

In other RA part-genres, for example, Xue and Ge [46] also conducted a cross-disciplinary and cross-rhetorical analysis of SC in RA abstract writing written by Chinese and native English writers within the field of applied linguistics. Using Hyland’s five-move model of RA abstracts, they found that native English writers strategically employed syntactic structures across rhetorical moves to effectively achieve different communicative purposes, and exhibited a balanced SC performance across whole abstract writing. Chinese writers, in contrast, showed a heavier reliance on coordinated structures, particularly in M1 *introduction* and M5 *conclusion*.

To date, while an expanding body of research has underscored the intricate relationship between rhetorical function and syntactic form in L2 academic writing [47], several important gaps remain. On the one hand, most studies integrating SC with rhetorical genre frameworks have primarily focused on cross-disciplinary variation, as previously discussed, leaving underexplored how writers from different L1 backgrounds may differ in their syntactic realization of rhetorical functions. On the other hand, existing research has largely concentrated on a limited range of RA part-genres, particularly abstract and introduction, due to the relative clarity of their rhetorical structures [18]. In contrast, other sections, especially the RA discussion, have received far less systematic attention, despite their crucial role as the interpretive core of academic argumentation.

To address these research gaps, the present study aims to examine SC variations across rhetorical move-steps in the RA discussion section written by Chinese and native English writers. Specifically, this study seeks to address the following research questions:

(1) Are there any differences between Chinese and native English writers in terms of SC across rhetorical move-steps in RA discussion scetion they produced? If yes, what are those differences?
(2) What underlying factors may explain the observed SC differences cross rhetorical move-steps?

## Methods

### The corpus

To examine the SC in the RA discussion, a corpus comprising two sub-corpora was constructed, totaling 200 RA discussion sections. Each sub-corpus contained 100 discussion sections written by either Chinese or native English writers. Hence, these two sub-corpora were referred to as CW and EW in short respectively.

The RA discussion sections were extracted from RAs which were selected randomly from five top-tier, peer-reviewed international journals within the field of applied linguistics, i.e., *Applied Linguistics*, *Journal of English for Academic Purposes*, *Second Language Writing*, *System*, and *Chinese Journal of Applied Linguistics*. Given the rigorous editorial and peer review processes, the RAs published in these journals are high-quality works. Therefore, the selection of these five journals guarantees the authenticity and representativeness of the language data used for the following research analysis. Moreover, clear sample selection criteria were established to ensure the consistency and rigor of the data collection process. Specifically, these criteria included: firstly, selecting RAs which are empirical studies within the field of applied linguistics. This choice was made to eliminate the potential influence of different disciplines and research types on SC performance [7,29]. Secondly, selecting RAs which have the IMRD macro genre structure (i.e., RAs have introduction, method, results, and discussion section) [20,21]. This choice was made to facilitate the extraction of the discussion section. Thirdly, selecting RAs published between 2018 and 2024, in order to capture linguistic features of “present-day” international academic prose [48]. Fourthly, selecting RAs written by either Chinese or native English scholars. In this thesis, scholars who have a Chinese name, obtained their bachelor’s and master’s degree from Chinese mainland universities, and are affiliated with institutions in mainland China are categorized as native Chinese writers. Similarly, scholars with English names who come from English-speaking countries such as the United States, United Kingdom, Canada, and Australia, and are affiliated with universities or institutions in these English-speaking countries at the same time, are categorized in the group of native English writers. For some scholars whose language background cannot be clearly determined, emails were sent to confirm their linguistic background. Lastly, selection was restricted to those RAs with a maximum of three authors (including the corresponding author). This choice was made to restrict the number of authors in those multi-authored RAs, allowing for better control of authors’ language backgrounds. Meanwhile, the author(s) of the selected RAs (including the first, second, and corresponding authors) have same language background (i.e., Chinese or English).

After selecting a sufficient number of RAs, their discussion sections were extracted. In the extraction process, section headings served as boundary markers [49]. For RAs with conventional section titles such as *Methods*, *Results*, and *Discussion*, the heading labels were employed as the main criteria for delineation. Additionally, for articles that featured content-specific headings such as *Missed positioning strategies in LG paper* (EW-42) or *Towards an understanding of feedback from the conceptual metaphors and their implied theoretical paradigms* (CW-58), additional confirmation was made to check their location in the article, as well as their content and actual rhetorical functions achieved to ensure that they indeed served as the discussion [47].

Irrelated content includes footnotes, figures, tables, and citation information in RA discussion texts were removed from the text. The final corpus comprised a total of 325,754 words. The word tokens of EW and CW corpus were almost equivalent, consisting of 164,805 and 160,949 words respectively. Detailed information about the corpus is provided in Table 1 below.

**Table 1:** Basic information of corpus.

Corpus	N	Tokens	Mean Length	Std. Deviation
Native English writers (EWs)	100	164,805	1,648.05	755.463
Native Chinese writers (CWs)	100	160,949	1,609.49	773.193

### Syntactic complexity measurement

To capture the SC performance across rhetorical move-steps in RA discussion section, this study adopted the set of fine-grained SC indices employed by Ziaeian and Golparvar [47], and further incorporated one global index, MLS (mean length of sentence), to provide a holistic measure of overall SC performance. In total, 18 SC indices were utilized, as summarized in Tables 2, 3, 4.

**Table 2:** Global-level SC metrics.

	Abbreviation Index label	Structure
1	MLS	Mean length of sentence

**Table 3:** Clausal-level SC metrics.

	Structure	Abbreviation Index label
1	dependents per clause	cl_av_deps
2	adverbial clauses per clause	advcl_per_cl
3	clausal complements per clause	ccomp_per_cl
4	clausal coordinating conjunctions per clause	cc_per_cl
5	conjunctions per clause	conj_per_clause
6	clausal subjects per clause	csubj_per_cl
7	nominal complements per clause	ncomp_per_cl
8	adverbial modifiers per clause	advmod_per_cl

**Table 4:** Phrasal-level SC metrics.

	Structure	Abbreviation Index label
1	dependents per nominal (no pronouns)	av_nominal_deps_NN
2	dependents per nominal subject (no pronouns)	av_nsubj_deps_NN
3	dependents per object of the preposition	av_pobj_deps_NN
4	adjectival modifiers per nominal (no pronouns)	amod_all_nominal_deps_NN_struct
5	prepositions per nominal (no pronouns)	prep_all_nominal_deps_NN_struct
6	possessives per nominal (no pronouns)	poss_all_nominal_deps_NN_struct
7	nouns as a nominal dependent per nominal (no pronouns)	nn_all_nominal_deps_NN_struct
8	relative clause modifiers per nominal (no pronouns)	rcmod_all_nominal_deps_NN_struct
9	(non-clausal) adverbial modifiers per nominal (no pronouns)	advmod_all_nominal_deps_NN_struct

Note: In the following sections, “NN_struct” and “(no pronouns)” have been omitted for the sake of brevity.

The selection of these indices was motivated by their sensitivity to rhetorical variation at different structural levels. Global indices (MLS) offer a holistic measure of sentence-level complexity [2,12,24,25], which is useful in distinguishing moves that require concise reporting of findings from those that involve elaborate commentary or argumentation [29,30,45]. Clausal indices (e.g., subordination ratio, dependent clauses per T-unit) capture the extent of clausal subordination, which is particularly relevant in move-steps where writers interpret or elaborate on theoretical and methodological details, since such rhetorical functions often demand in using structures with elaborative functions (e.g., non-finite dependent clause) [29]. Phrasal indices (e.g., complex nominals per clause, prepositional phrase density) reflect the use of information-condensed structures of NPs, which are especially common in those “propositionally dense” move-steps where writers account results, claim centrality, or highlight significance [29,30]. Moreover, these SC indices are sensitive to measure cross-linguistic syntactic variations in RA writing as well [47]. By adopting this multidimensional set of SC indices, the present study ensures that all SC dimensions were captured (i.e., global-level, clausal-level, and phrase-level complexity) [27].

We employed TAASSC (*Tool for the Automatic Assessment of Syntactic Sophistication and Complexity*, version 1.3.8) [50], which is capable of providing a broad range of fine-grained SC indices with established reliability and validity [32,33,47], to analyze these SC indices across the rhetorical move-steps of the RA discussion in present study.

### Annotation of rhetorical move-steps

To analyze the SC across different rhetorical move-steps, we used MAXQDA to annotate rhetorical move-steps manually. The annotation process was conducted manually, in accordance with the hierarchical seven-move framework proposed by Yang and Allison [49] for discussions in RAs, as presented in Table 5. We acknowledge that manual coding process is prone to errors and may introduce subjective interpretation [26], which may affect the categorization of borderline cases. To minimize such potential drawbacks and bias, we established clear coding guidelines prior to annotation, discussed ambiguous cases, and ensured independent coding by two researchers [5].

**Table 5:** Seven-move framework of RA discussion section.

Rhetorical Move-Steps	Tag	EW		CW	
		Count^a^	Percentage^b^	Count	Percentage
Move 1—Background information	M1	430	6.06%	295	5.25%
Move 2—Reporting results	M2	915	12.90%	870	15.48%
Move 3—Summarizing results	M3	385	5.43%	195	3.47%
Move 4—Commenting on results^*^	M4				
Move 4 Step 1—Interpreting results	M4S1	2055	28.96%	1535	27.31%
Move 4 Step 2—Comparing results with literature	M4S2	735	10.36%	765	13.61%
Move 4 Step 3—Accounting for results	M4S3	530	7.47%	680	12.10%
Move 4 Step 4—Evaluating results	M4S4	470	6.62%	280	4.98%
Move 5—Summarizing the study	M5	145	2.04%	115	2.05%
Move 6—Evaluating the study	M6				
Move 6 Step 1—Indicating limitations	M6S1	260	3.66%	185	3.29%
Move 6 Step 2—Indicating significance/advantage	M6S2	170	2.40%	115	2.05%
Move 6 Step 3—Evaluating methodology	M6S3	200	2.82%	120	2.14%
Move 7—Deductions from the research	M7				
Move 7 Step 1—Making suggestions	M7S1	125	1.76%	120	2.14%
Move 7 Step 2—Recommending further research	M7S2	315	4.44%	190	3.38%
Move 7 Step 3—Drawing pedagogic implication	M7S3	360	5.07%	155	2.76%

^a^‘Count’ represents the total number of sentences used by different writers in this move.

^b^^‘^Percentage’ represents the ratio of sentences in certain rhetorical move relative to the total number of sentences.

Additionally, 20% (n = 40) of the texts were randomly selected and annotated by a senior professor with extensive experience in EFL writing analysis. The professor was provided with detailed training specifying the rhetorical framework and our annotation guidelines. This procedure was implemented to further validate the reliability of the annotation results. All disagreements were resolved through discussion, resulting in a high Krippendorff’s alpha (94.7%).

### Research procedures

The normality of the data was tested using the Shapiro-Wilk test, and the results indicated that all SC indices significantly deviated from a normal distribution. Therefore, the non-parametric Mann-Whitney U test was employed as the statistical method in this study. A series of Mann-Whitney U tests was then conducted to examine whether Chinese and native English writers exhibited significant SC differences across rhetorical move-steps in RA discussion sections. To visualize the overall patterns of SC variation across rhetorical move-steps, we created a heatmap using the p-values obtained from the U tests. Although p-values do not provide effect size information, they were used here as an intuitive indicator of where statistically significant contrasts occurred between groups. To enhance the visual contrast and make small p-values more distinguishable, significant p-values (p < .05) were log-transformed using the Excel formula ‘*=LN(number)*’. Because log transformation requires positive values and because our primary aim was to highlight significant versus non-significant results rather than subtle differences among the latter, all non-significant p-values (p ≥ .05) were assigned a value of 1 prior to log-transformation. This procedure allowed the heatmap to clearly differentiate significant findings while avoiding distortions caused by very small numerical values.

## Results

**Fig 1:**
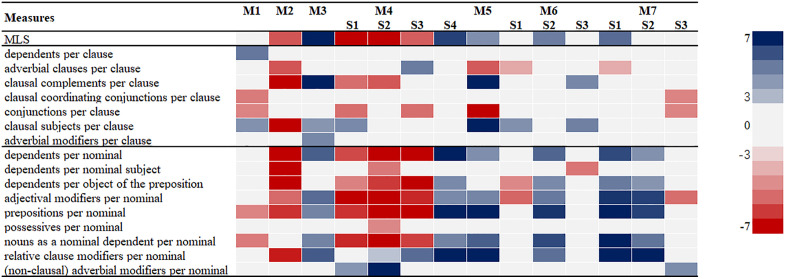
Significant differences across rhetorical move-steps. Note. Red represents the mean rank score of CW is significantly higher than that of EW, whereas blue indicates the opposite.

The results of the Mann-Whitney U tests, as shown in Table 6, revealed significant SC differences between the two groups of writers across rhetorical move-steps in the RA discussion section (Detailed results are available in the supplementary materials). The heatmap (Fig 1) offers a clearer and more intuitive illustration of SC differences between groups across rhetorical move-steps, with red indicating significantly higher mean rank values for Chinese writers and blue indicating higher values for native English writers.

**Table 6:** U-test results of SC in different rhetorical move-steps.

SC Measures	M1	M2	M3	M4				M5	M6			M7		
				S1	S2	S3	S4		S1	S2	S3	S1	S2	S3
MLS		**	***	***	***	*	**	**		*		*		
dependents per clause	*													
adverbial clauses per clause		*				*		*	*			*		
clausal complements per clause		***	***	*	*			***			*			
clausal coordinating conjunctions per clause	*													*
conjunctions per clause	*			*		*		***						*
clausal subjects per clause	*	***	*	*				***	**		*			
adverbial modifiers per clause			*											
dependents per nominal		***	**	**	***	***	***	*		**		**	*	
dependents per nominal subject		***			*						*			
dependents per object of the preposition		***		*	**	***	*		*	*		*	*	
adjectival modifiers per nominal		*	*	***	***	**	*	*	*	*		**	**	*
prepositions per nominal	*	**	*	**	***	***	***	***		**		***	**	
possessives per nominal					*									
nouns as a nominal dependent per nominal	*		*	**	***	**	*	**		**		***	*	
relative clause modifiers per nominal		**	**			*	***	***		**		***	***	
(non-clausal) adverbial modifiers per nominal				*	***									*

Note: MLS: Mean length of sentences;

* *p* < 0.05; ** *p* < 0.01; *** *p* < 0.001.

Overall, the findings indicate that Chinese and native English writers demonstrated distinct SC patterns across multiple rhetorical move-steps. This suggests that, in addition to disciplinary factors [29,30,45], L1 background also shapes writer’s SC performance in fulfilling certain rhetorical functions. This observation is consistent with the theoretical underpinnings of **contrastive rhetoric**, which holds that writers’ L1 and cultural background shape their discourse organization and syntactic choices in L2 writing [22]. With respect to global complexity, Chinese writers produced significantly higher MLS values than their native English counterparts in M2 *Reporting results*, M4S1 *Interpreting results*, M4S2 *Comparing results with literature*, and M4S3 *Accounting for results*. In contrast, their MLS values were significantly lower in M3 *Summarizing results*, M4S4 *Evaluating results*, M5 *Summarizing the study*, M6S2 *Indicating significance/advantage*, and M7S1 *Making suggestions*. Taken together, these global SC patterns suggest that Chinese writers showed a salient preference: in those result-reporting move-steps (e.g., reporting, comparing, and accounting), they tend to construct longer sentences than native English writers, whereas in the stance-advancing move-steps (e.g., summarizing, evaluating, highlighting significance, and making suggestions), they more likely opting comparatively shorter sentences.

However, examining differences only at the global level does not sufficiently reveal how SC was performed across specific move-steps. To gain a more nuanced understanding, it is necessary to also consider clausal- and phrasal-level measures. Building on the SC patterns illustrated in Fig 1, we further classified those rhetorical move-steps that presented similar SC patterns into three groups: (1) The group of rhetorical move-steps in which Chinese writers exhibited significantly greater SC; (2) The group of rhetorical move-steps in which native English writers exhibited significantly greater SC; and (3) The group with few significant between group SC differences.

### The group of rhetorical move-steps in which Chinese writers exhibited significantly greater SC

The first group comprised the rhetorical move-steps in which CWs exhibited significantly higher values across multiple SC indices in all three dimensions compared to their native English counterparts. Specifically, these move-steps included M2 *Reporting Results*, M4S1 *Interpreting results*, M4S2 *Comparing results with literature*, and M4S3 *A*ccounting for results**. The results indicated that CWs consistently achieved higher mean rank scores than EWs in these move-steps in terms of the following SC indices: MLS, dependents per nominal, dependents per object of the preposition, adjectival modifiers of nominals, and prepositions per nominal (see Table 7; detailed results are available in the supplementary material).

**Table 7:** Results of SC indices with significant differences in specific rhetorical move-steps (CW Group).

SC Measures	M2	M4S1	M4S2	M4S3
MLS	.010^**^	<.001^***^	<.001^***^	.014^*^
dependents per nominal	<.001^***^	.008^**^	<.001^***^	<.001^***^
adjectival modifiers per nominal	.020^*^	<.001^***^	<.001^***^	.003^**^
prepositions per nominal	.004^**^	.004^**^	<.001^***^	<.001^***^
dependents per object of the preposition	<.001^***^	.039^*^	.005^**^	<.001^***^

Note: MLS: Mean length of sentences;

* *p* < 0.05; ** *p* < 0.01; *** *p* < 0.001.

In addition to producing longer MLS values in these move-steps, CWs employed significantly more dependents in their NP constructions than EWs. Specifically, the high number of nominal dependents was primarily attributed to the following three phrasal dependent NP indices, i.e., adjectival modifiers, prepositions, and dependents per object of the preposition. Based on our observation of the CW corpus, we found that CWs favored in using longer pre-modifier sequences in NPs. This was also evidenced by the markedly higher value of adjectival modifiers per nominal. As shown in Example 1, complex NPs are enclosed in square brackets, with the head nouns highlighted in bold. The average pre-modifier length of complex NPs produced by CWs surpassed that of their native English counterparts (Mean pre-modifier length of NPs_CW_ = 1.4; Mean pre-modifier length of NPs_EW_ = 0.875). This tendency to employ longer pre-modifiers in NPs is consistent with findings reported by Cao and Xiao [36]’s study, and may be attributed to the influence of language transfer, reflecting the significant structural discrepancies between Chinese and English. Unlike English, which typically places modifiers after the head noun, Chinese adopts a “modifying-modified” word order [14] and does not have post-modifiers.

**Example 1:**

(a) [Another **explanation**] for [the **results** of this study] is that learners are in general more accustomed to [oral **feedback**] than [text-based **feedback**] in [language **classrooms**] and this facilitated [learners’ **understanding** of [audio-based **CF** that they received]] compared to [text-based **CF**]. (EW-10-M4S3)
(b) [One possible **explanation**] is that [the EFL **subjects** in [the aforementioned **studies**]] were [college **students** with [little academic writing **experience**]], while [the **subjects** in this study] are [doctoral **students** with [a longer **experience**] of learning/using English and with [extensive **exposure**] to [academic written **texts** in English]]. (CW-8-M4S3)

Meanwhile, CWs presented higher mean rank scores in another two phrasal SC indices: prepositions per nominal and dependents per object of the preposition. In these rhetorical move-steps, CWs showed a preference for employing prepositional phrases as NP post-modifiers, particularly through multiple embedded prepositional phrases that create intricate hierarchical meaning relations within a single NP [6].

In Example 1(b), the NP “doctoral students ***with*** a longer experience ***of*** learning/using English and ***with*** extensive exposure ***to*** academic written texts in English” contains two *with*-phrases coordinated by *and*. The first *with*-phrase embeds an *of*-phrase (*of learning/using English*), while the second *with*-phrase embeds a *to*-phrase (*to academic written texts)* which further includes an *in*-phrase (*in English*). By using such embedded prepositional phrases, CWs constructed compressed NP structures with multi-layered meanings, thereby compacting additional information related to the head noun (*students*). The frequent use of such multiple prepositional phrases as post-modifiers among CWs was partly due to their developed awareness of the need to use phrasal-level structures to achieve a condensed discourse style in academic writing [2,5,6,14,26–28,51]. Therefore, it was evident that the significantly longer MLS observed in these rhetorical move-steps by CWs could be attributed to the frequent use of these complex NP structures compared to EWs.

**Example 2:**

[The findings, ***which*** align with Smith’s assertion that genre-specific conventions influence SC], further corroborate [the importance of [disciplinary variation in [academic writing]]]. (EW-23-M4S2)

However, it should also be noted that not all types of NP modifiers were frequently used by CWs. Relative clause modifiers represented the only type of nominal dependent in which CWs scored significantly lower than the EWs. As shown in Table 7, relative clauses functioning as NP modifiers in M4S1 *Interpreting results* and M4S2 *Com*paring results with literature** exhibited an opposite trend compared with the other nominal indices. For instance, in Example 2, the relative clause “*which aligns with Smith’s assertion that genre-specific conventions influence SC*” functioned as a NP modifier, providing additional interpretive information to the head noun “*The findings*”. However, CWs rarely employed such clausal modifiers when interpreting or comparing previous studies.

### The group of rhetorical move-steps in which native English writers exhibited significantly greater SC

EWs significantly outperformed their Chinese counterparts and exhibited a similar SC pattern in several rhetorical move-steps, particularly M3 *Summarizing results*, M4S4 *Evaluating results*, M5 *Summarizing the study*, M6S2 *Indicating significance/advantage*, M7S1 *Making suggestions*, and M7S2 *Recommending further research*. As shown in Table 8, these move-steps consistently displayed clear contrasts in SC performance between the two writer groups. In addition to significantly lower MLS, CWs employed dependents per nominal, adjectival modifiers per nominal, prepositions per nominal, nouns as a nominal dependent per nominal, and relative clause modifiers per nominal less frequent than the EWs. Similar to the first group, these significant differences were also concentrated in phrasal-level complexity.

**Table 8:** Results of indices with significant differences in specific rhetorical move-steps (EW Group).

SC Measures	M3	M4S4	M5	M6S2	M7S1	M7S2
MLS	<.001^***^	.003^**^	.038^*^	.022^*^	.012^*^	–
dependents per nominal	.008^**^	<.001^***^	.038^*^	.002^**^	.004^**^	.042^*^
adjectival modifiers per nominal	.012^*^	.032^*^	.028^*^	.019^*^	.002^**^	.003^**^
prepositions per nominal	.026^*^	<.001^***^	<.001^***^	<.002^**^	<.001^***^	.003^**^
nouns as a nominal dependent per nominal	.021^*^	.026^*^	.008^**^	.004^**^	<.001^***^	.016^*^
relative clause modifiers per nominal	.007^**^	<.001^***^	<.001^***^	.014^*^	<.001^***^	<.001^***^

Note: MLS: Mean length of sentences;

* *p* < 0.05; ** *p* < 0.01; *** *p* < 0.001.

**Example 3:**

(a) [The **significance** of this study] lies in [its comprehensive **analysis** of [phrasal **structures**]], which not only enhances [our **understanding** of [linguistic **complexity** in [academic **writing**]]] but also offers [valuable **insights**] for improving [pedagogical **approaches** in [language **education**]]. (EW-26-M6S2)
(b) [This study is significant as it provides [a detailed **analysis** of IDL], deepening [our **understanding** of [linguistic **complexity**]] and contributing [valuable **insights**] for [L2 oral **performances**]. (CW-54-M6S2)

**Example 4:**

(a) [The basic **information** of [the RA **corpora**]] is summarized in nouns. And [declarative **sentences**] are overwhelmingly dominant. (CW-8-M3)

In sum, we ﬁnd that although [strategy **instruction**] can increase [strategy **use**], and factors such as [task **type**], proﬁciency, and [**perception** of collaboration] have been shown to affect the [**frequency** of [strategy **use**]].(EW-19-M3)

These results suggest that CWs tended to construct nominal structures with fewer phrasal dependents than EWs in these rhetorical move-steps. Examples 3(a) and 3(b) illustrate this SC pattern within M6S2 *I*ndicating significance/advantage of the research**. In Example 3(a), written by a native English writer, has nine complex nominals (marked in brackets), five of which have prepositional phrases as post-modifiers. In contrast, Example 3(b), written by a Chinese writer, contains nominals with fewer modifiers than that of EWs. Accordingly, we assumed that the longer sentences produced by EWs in these rhetorical move-steps may primarily result from their denser use of both NPs and their phrasal dependents. The syntactic structures used by CWs in these move-steps were less complex compared to their native English counterparts, as illustrated in Example 4 extracted from M3 *Summarizing results*. It is evident that 4(a), written by a CW, has a relatively simple syntactic structure with only three NPs, while 4(b), written by an EW, contains both clausal subordination and coordination, with six NPs, significantly more than in 4(a). These results echo findings previous SC studies on L2 academic writing in that SC variation between L1 and L2 academic writing is more pronounced at the phrasal level than at the clausal level, and SC differences at phrasal-level are more salient in L2 academic writing than clausal complexity [23].

### The group of move-steps with few differences

In the third group, the rhetorical move-steps did not demonstrate consistent patterns of SC variation and have few significant differences between CWs and EWs. These move-steps included M1 **Background informatio*n*, M6S1 *Indicating limitations*, M6S3 *Evaluating methodology*, and M7S3 *Drawing pedagogic implication*. The findings suggest that the two groups displayed broadly comparable SC performance in these rhetorical contexts.

## Discussion

The findings of present study shed light on the different SC patterns across rhetorical move-steps in RA discussion section written by Chinese and native English writers. The distinct SC patterns between the two writer groups revealed in present study indicate that the L1 background indeed influences their SC performance across rhetorical move-steps in RA discussion. It is evident that the significant differences are primary concentrated on the phrasal-level complexity. These results provide additional support for previous studies that revealed considerable differences at the phrasal-level in dense academic writing styles between CWs and EWs [14,52].

In the first group of rhetorical move-steps, CWs exhibited significantly greater SC contributed by phrasal complexity compared to their native English counterparts in the following rhetorical move-steps: M2 *Reporting Results*, M4S1 *Interpreting results*, M4S2 *Comparing results with literature*, and M4S3 *Accounting for results*. This suggests that the NPs produced by CWs in these move-steps were structurally more elaborate, characterized by more frequent use of extended pre-modifier sequences and multi-layered prepositional phrases. This distinct SC pattern can be linked to the different rhetorical strategies employed by writers to fulfill these rhetorical functions. From a functional perspective, these essential rhetorical move-steps necessitate a deep analysis and interpretation of complex relationships and multiple informational layers, offering detailed arguments and supporting evidence [49]. To effectively accomplish these communicative goals of commenting on the results, writers typically need to employ intricate phrasal structures to precisely convey the interpretations, comparisons, and explanations involved [49,47]. Specifically, they need to elucidate the meaning and significance of the findings, establish connections with existing literature, and offer insights into what the current data reveals. Meanwhile, they must provide explanations for the results obtained. In these essential rhetorical move-steps, writers are required to deeply analyze and interpret intricate relationships and multi-layered information, providing detailed arguments and robust evidence to support their findings [49]. Faced with these rhetorical requirements, CWs show a tendency to condense complex relationships into densely packed NPs, which is consistent with academic writing norms that emphasize brevity and information density [6,48]. In contrast, EWs demonstrate a greater degree of syntactic flexibility to meet these rhetorical goals. For instance, in M4S2, many EWs chose clausal modifiers to link the present findings with previous literatures. This observation aligns with Lu, Casal and Liu’s [30] finding that sentences used in reviewing previous research contain significantly higher clausal subordination [53]. The current finding suggests that EWs, benefiting from their superior linguistic abilities as natives, can adaptively employ a variety of syntactic resources to articulate their arguments in RA discussion writing.

In the second group of rhetorical move-steps where the SC pattern was reversed: CWs displayed significantly lower NP complexity compared to EWs in M3 *Summarizing results*, M4S4 *Evaluating results*, M5 *Summarizing the study*, M6S2 *Indicating significance/advantage*, M7S1 *Making suggestions*, and M7S2 *Recommending further research*. This discrepancy indicates that Chinese scholars can grasp the main and obligatory rhetorical goals of the discussion section, such as reporting (M2) and commenting on results (M4), but occasionally overlook other move-steps deemed optional [49]. In M3 *Summarizing results* and M5 *Summarizing the study*, many RAs’ discussions in the CW corpus lacked the sentences achieving these moves (as evidenced by the frequency data in Table 5), where both the number and proportion of sentences for these moves were much lower compared to other rhetorical move-steps and those in the EW group. This is partially influenced by Chinese academic writing conventions, which have different rhetorical organizations and writing norms compared to international academic writing in English [54–56]. Therefore, CWs’ comprehension of move-steps and rhetorical structure of RA discussion may vary from international-oriented RA writing. For novice L2 academic writers seeking to engage with the global academic community, grasping the full rhetorical structure of RA discussions and adapting to the differing writing conventions can pose a considerable challenge. Moreover, from a functional perspective, for M6S2 *Indicating significance/advantage*, M7S1 *Making suggestions,* and M7S2 *Recommending further research*, these carry high-risk rhetorical functions where writers are required to explicitly express their stance in front of the potential readerships on the one hand. It can be assumed that CWs adopt a more cautious approach to avoid potential criticism from readers. On the other hand, these move-steps are inherently more subjective compared to others, posing a notable challenge for L2 writers to balance subjectivity and the objectivity required in academic writing. From a cognitive complexity perspective, achieving these rhetorical functions requires greater cognitive effort compared to moves such as M1 *Background information* and M2 *Reporting results*, whose communicative goals are more descriptive in nature. As earlier studies have shown, the cognitive task complexity inherent in different genres can influence the choice and performance of linguistic complexity in L2 writing [11,57], and we believe this also applies across various rhetorical move-steps. Therefore, akin to M3, M4S4, M5, CWs have similarly chosen to reduce the number of sentences in realizing these rhetorical move-steps. Additionally, the SC performance in these rhetorical move-steps is also influenced by cultural and language factors. CWs exhibited significantly lower use of clausal modifiers (relative clauses) across various rhetorical moves (except M2) compared to EWs. This consistent pattern of lower usage can largely be attributed to language transfer, as the syntactic structures of Chinese and English differ substantially. In Chinese, the structure of relative clauses is relatively simple, connecting the main clause and modifier with the particle ***de***, in contrast to English, where relative clauses are typically introduced by diverse relativizers such as *who*, *which*, and *that*. Moreover, when encountering rhetorical move-steps that are less familiar or less conventional in Chinese academic writing, some authors may draw more heavily on L1-based syntactic pattern or translation in constructing the optional move-steps to bridge the obligatory ones, a tendency that is especially pervasive among novice L2 writers. This process may lead to increased use of syntactic patterns characteristic of L1 or simplified sentence structures [42, 58], ultimately yielding less native-like usage of NPs or clause structures. The present results provide empirical evidence regarding language transfer in L2 academic writing [14,36].

Moreover, in these stance-taking rhetorical move-steps, Chinese writers appear to adopt more conservative syntactic strategies than their native English counterparts. Under the constrained language framework [38], such restraint in syntactic elaboration may serve as a risk-avoidance mechanism in socially and cognitively demanding academic contexts. In these high-stakes rhetorical contexts, CWs may limit the use of complex structures to reduce potential errors and manage face-threatening discourse, thereby reflecting a typical feature of constrained language varieties: utilizing limited syntactic resources to fulfill rhetorical functions effectively. Furthermore, Chinese NP construction conventionally follows a “modifying-modified” pattern from a typological perspective. This ingrained L1 structure may transfer into L2 English writing, leading CWs to favor pre-modifier sequences (e.g., adjectival modifier sequences) at the expense of embedding clausal dependents such as relative clauses which are more structurally more complex and resource-intensive. This tendency aligns with the constrained language framework, where L2 writers prefer structurally simpler phrasal strategies over clausal complexity due to different cognitive and linguistic load, especially when executing epistemically and socially sensitive rhetorical move-steps. From cultural perspective, the differences in SC may reflect underlying Chinese cultural traditions. Specifically, it appears to be shaped by the Chinese cultural tradition of modesty, which emphasizes humility and avoidance of overt self-promotion [59]. This cultural norm influences CWs to refrain from explicitly presenting and highlighting their contributions and achievements in academic writing, particularly when discussing the significance of their research (M6S2 *Indicating significance/advantage*) or providing constructive suggestions and viewpoints (M7S1 *Making suggestions* and M7S2 *Recom*mending further research**). As a result, CWs adopt a restrained strategy in their writing on these moves, which explains the observed SC differences.

The minimal differences observed in the third group of move-steps (e.g., M1 *Background information* and M6S1 *Indicating limitations*) may be attributed to their highly conventionalized rhetorical functions. These move-steps typically rely on formulaic linguistic patterns, such as presenting established knowledge or explicitly stating study limitations, which are widely shared across academic discourse communities. As a result, both Chinese and native English writers tend to adopt similar syntactic strategies, leading to fewer measurable differences in SC. From a constrained language perspective [38], these move-steps impose relatively low rhetorical demands, requiring fewer syntactic resources to accomplish communicative goals. Moreover, disciplinary conventions in applied linguistics, as well as the limited corpus scope of this study, may also have attenuated potential cross-linguistic variation.

### Pedagogical implications

The present study provides valuable pedagogical implications for the L2 academic writing, especially for RA discussion writing. Firstly, L2 academic writing instruction should pay more attention to the micro-level aspects of academic writing (i.e., rhetorical structure), allowing novice learners to gain a basic understanding of rhetorical structures in academic writing practice. For example, instructors can draw on corpora to deliver genre-based instruction, using authentic RA writing examples by advanced international writers to guide L2 novice learners how to use different types of syntactic structures in genre-appropriate and functionally effective ways. For example, native writers tend to employ relative clause as NP modifier as effective resources for *Comparing results with literatures* and *Accounting for results* in their discussion writing.

Moreover, it is crucial to recognize that the relationship between surface syntactic structures and rhetorical functions in academic writing are not fixed. This variability underscores the need to provide explicit guidance on linguistic choices in RA writing for L2 novice writers. For example, instructors can integrate comparative analysis of RAs authored by native and L2 writers into writing instruction. By guiding learners to examine and compare the syntactic choices employed by different writer groups, learners not only can obtain the awareness that language and cultural background will influence the use of linguistic recourses in academic writing, but also can acquire knowledge of the linguistic structures that are conventionally used in academic writing practice.

Additionally, Table 9 ranks rhetorical move-steps in the discussion section by the magnitude of SC variation — from the least to the most pronounced. This ranking suggests that, for internationally oriented L2 writers (particularly Chinese writers), those move-steps showing greater SC variation warrant prioritized instructional focus. Instructors can prioritize these “difficult” move-steps through explicit, targeted instruction. For instance, they can scaffold learners’ engagement with these problematic move-steps by first present the genre-appropriate use of syntactic structures, then guiding students through structured draft-writing activities focused on these move-steps, and finally prompting independent reflection and revision of their drafts with reduced support.

**Table 9:** Ranking of rhetorical move-steps by difficulty of acquisition.

Easiest	Middle	Challenging
M1Background information	M2Reporting results	M3Summarizing results
M6S1 Indicating limitations	M4S1 Interpreting results	M4S4 Evaluating results
M6S3 Evaluating methodology	M4S2 Comparing results with literature	M5 Summarizing the study
M7S3 Drawing pedagogic implication	M4S3 Accounting for results	M6S2 Indicating significance/ advantage
		M7S1 Making suggestions
		M7S2Recommending further research

## Conclusions

In this study, we employed a corpus-based approach to examine the SC differences across different rhetorical move-steps in RA discussion sections written by Chinese and native English writers. Our findings reveal significant SC differences between two writer groups in different move-steps, with the most pronounced disparities primarily concentrating in phrasal-level complexity. These findings may advance our understanding of the relationship between SC and rhetorical functions in L2 academic writing, offering insights into how linguistic structures are strategically employed by L2 writers to achieve specific communicative purposes.

Nevertheless, we also acknowledged that this study has several limitations. Firstly, it relied on a specific corpus of RAs within the field of applied linguistics. Consequently, the findings may have limited applicability to other fields or writing genres. For future research, it is recommended to broaden the scope of analysis to include a wider range of disciplines and writing genres. Secondly, this study only selected a small set of representative SC indices. This may have limited the scope of syntactic features captured, and we suggest future studies can employ a broader range of SC indices (such as L2SCA indices, and other fine-grained indices) to provide a more comprehensive account of the relationship between SC and rhetorical structures. Thirdly, the annotation of rhetorical move-steps was conducted manually, which, as noted by one reviewer, may introduce potential subjective bias. Future research could consider incorporating semi-automatic or fully automatic annotation tools to improve reliability and consistency. Additionally, while this study briefly considered cultural influences, as one reviewer pointed out, the lack of empirical evidence restricts the explanatory power. Future studies can strengthen this aspect by incorporating qualitative methods such as interviews or questionnaires to triangulate the quantitative findings.

In conclusion, this study highlights the importance of integrating SC with rhetorical analysis in understanding L2 academic writing. By shedding light on how linguistic and rhetorical factors interact, it not only contributes to theoretical perspectives on L2 writing but also provides pedagogical implications for genre-based writing instruction.
